# Supplementary material for: NCOA4 drives ferritin phase separation to facilitate macroferritinophagy and microferritinophagy
Source: J Cell Biol. 2022 Sep 6;221(10):e202203102. doi: 10.1083/jcb.202203102 (PMC9452830; doi:10.1083/jcb.202203102)
Supplement: Table S1 — lists primers used for plasmid construction. [file JCB_202203102_TableS1.docx]

**Table S1. Primers used for plasmid construction**

Name Sequence (5' to 3')

NCOA4KO-1F cttgtggaaaggacgaaacaccggtcttagaagccgtgaggtagttttagagctagaaatagcaag

NCOA4KO-2R cttgctatttctagctctaaaactacctcacggcttctaagaccggtgtttcgtcctttccacaag

NCOA4KO-3F ccatgattacgccaagctagcatgccAAGgctgataaacttgtaaaaccgtagg

NCOA4KO-4R gcggtgggctctatggataAAGttaTACCTCACGGCTTCTAAGACATTCC

NCOA4KO-5F GTATCTTATCATGTCTGGATCtttGGAtaaGACCTTATTTATCAGCTTAAAGAGG

NCOA4KO-6R ccagtgagttcgagcttgcatgccGGAagtataatattaaatacatgaagc

NCOA4KO-7F GTATCTTATCATGTCTGGATCtttGGAtaaccctttttgcgtattttaacttttg

NCOA4KO-8R ccagtgagttcgagcttgcatgccGGAttctcaagaaagtcactattcatagc

TAX1BP1KO-1F cttgtggaaaggacgaaacaccgGTTCTGTTACGTTACCCATAgttttagagctagaaatagcaag

TAX1BP1KO-2R cttgctatttctagctctaaaacTATGGGTAACGTAACAGAACcggtgtttcgtcctttccacaag

TAX1BP1KO-3F ccatgattacgccaagctagcatgccAAGgggtcctaaagcttatttttacc

TAX1BP1KO-4R ggatgcggtgggctctatggataAAGcaaaattattaaacttcctattataaaattc

TAX1BP1KO-5F GTATCTTATCATGTCTGGATCtttGGAagatagtgattcattgataattttaag

TAX1BP1KO-6R ccagtgagttcgagcttgcatgccGGAggccaaaagatttttttaagcatttc

mGFP-1F gggccatcgatataaagcttgccaccATGGTGAGCAAGGGCGAGGAG

mGFP-2R gggccgcggccgcttactcgagacccgaattcccggatcccgagcctgaaccCTTGTACAGCTCGTCCATGCC

mRuby3-1F ccggatctgccatcgatataaagcttgccaccATGGTGAGCAAGGGCGAGGAG

mRuby3-2R gggccgcggccgcttactcgagacccgaattcccggatcccgagcctgaaccCTTGTACAGCTCGTCCATGCC

FTH1-1F ggttcaggctcgGGATCCggGAATTCgACGACCGCGTCCACCTCGCAGG

FTH1-2R tttacgtaGCGGCCGCtCTCGAGTTAGCTTTCATTATCACTGTC

FTL-1F ggttcaggctcgGGATCCggGAATTCgAGCTCCCAGATTCGTCAGAATT

FTL-2R tttacgtaGCGGCCGCtCTCGAGTTAGTCGTGCTTGAGAGTGAGCC

NCOA4-1F ggttcaggctcgGGATCCggGAATTCgAATACCTTCCAAGACCAGAG

NCOA4-2R CCGGATCcGCTCGAGacccGTCGAGCTACATCTGTAGAGGAGTTCG

TAX1BP1-1F ggttcaggctcgGGATCCggGAATTCgACATCCTTTCAAGAAGTCCC

TAX1BP1-2R ggaatttacgtaGCGGCCGCtTTACTCGAGCTAGTCAAAATTTAGAACATTCTG

muGFP-NCO-1F gaataaacacacataaacaaacaaaAAGCTTgccgccaccATGGTGAGCAAGGGCGAGGAGCTG

muGFP-NCO-2R GCTGTACAAGggttcaggctcgGGATCCggtaattcgAATACCTTCCAAGACCAGAGTGG

muGFP-NCO-3F GGAAGGTATTcgaattaccGGATCCcgagcctgaaccCTTGTACAGCTCGTCCATGC

muGFP-NCO-4R cgatttcaattcaattcaatGGATCCTTACATCTGTAGAGGAGTTCGATATAACC

FTH1-yeast-1F cgaataaacacacataaacaaacaaaAAGCTTgccgccaccATGACGACCGCGTCCACCTCGCAGG

FTH1-yeast-2R cgatttcaattcaattcaatGGATTAGCTTTCATTATCACTGTCTCCCAGG

Y2H-NCO-1F ggttcaggctcgGGATCCggGAATTCgAATACCTTCCAAGACCAGAG

Y2H-NCO-2R ggaatttacgtaGCGGCCGCtTTACTCGAGtcaTTATCTCTTCTCCAGGAAGGGCCC

NCO-dN-1F ggttcaggctcgGGATCCggGAATTCgGAGCACTTGATGGCTCATGC

NCO-dN-2R ggaatttacgtaGCGGCCGCtCTCGAGCTACATCTGTAGAGGAGTTCG

NCO-dIDR1-1F GGCTGTATCTCCATGCCACGTGAAACttcTGAaAAaTTc

NCO-dIDR1-2R gAAtTTtTCAgaaGTTTCACGTGGCATGGAGATACAGCC

NCO-dM-1F GCCTGAGAATGGCAGTAAGGATAAAAATGGGATGC

NCO-dM-2R GCATCCCATTTTTATCCTTACTGCCATTCTCAGGC

NCO-dIDR2-1F GGCTTCTGAAGAAAGAAGGATCCTGGTGTTCCTTTAAC

NCO-dIDR2-2R GTTAAAGGAACACCAGGATCCTTCTTTCTTCAGAAGCC

NCO-dC-1F ggttcaggctcgGGATCCggGAATTCgAATACCTTCCAAGACCAGAG

NCO-dC-2R ggaatttacgtaGCGGCCGCtCTCGAGCTAAGTATTCATGGGGCTTTTAAAC

NCO-I56E-1F CTCAGATTCACAGTTGCgaaAGCCGTCACCTGGAATG

NCO-I56E-2R CATTCCAGGTGACGGCTttcGCAACTGTGAATCTGAG

NCO-L63R-1F CCGTCACCTGGAATGTagaAGAAGCCGTGAGGTATG

NCO-L63R-2R CATACCTCACGGCTTCTtctACATTCCAGGTGACGG

AAVS-Tet-On-1F gtgagttcgagcttgcatgccGGATCCcaccacgtgatgtcctctgagc

AAVS-Tet-On-2R GGCCAAAGTGGATCTCTGCTGTCCCTGGGATCCtggggtggaggggacagataaaagtacc

AAVS-Tet-On-3F ggtacttttatctgtcccctccaccccaGGATCCCAGGGACAGCAGAGATCCACTTTGGCC

AAVS-Tet-On-4R gtaaccattataagctgcaataaacaagttgcggccgctctcgagtaattccaattctccaggcgatctgacgg

AAVS-Tet-On-5F ccgtcagatcgcctggagaattggaattactcgagagcggccgcaacttgtttattgcagcttataatggttac

AAVS-Tet-On-6R gggcgatgtgcgctctgccctatGATCCAGACATGATAAGATACATTGATGAGTTTGG

AAVS-Tet-On-7F gcggtgggctctatggataAAGCTTgacaggattggtgacagaaaagcc

AAVS-Tet-On-8R gattacgccaagctagcatgccAAGCTTgggagtagaggcggccacgacc

mR-RAB5QL-1F CCGTCAGATCGCCTGGAGAATTGGAATTAATGGTGAGCAAGGGCGAGGAGCTCATCAAGG

mR-RAB5QL-2R GCTGCAATAAACAAGTTGCGGCCGCtTCAGTTACTACAACACTGGCTTCTGGC
