# Supplementary figures and images for: NCOA4 drives ferritin phase separation to facilitate macroferritinophagy and microferritinophagy
Source: J Cell Biol. 2022 Sep 6;221(10):e202203102. doi: 10.1083/jcb.202203102 (PMC9452830; doi:10.1083/jcb.202203102)

anti-FTH1 (Figure 1C)

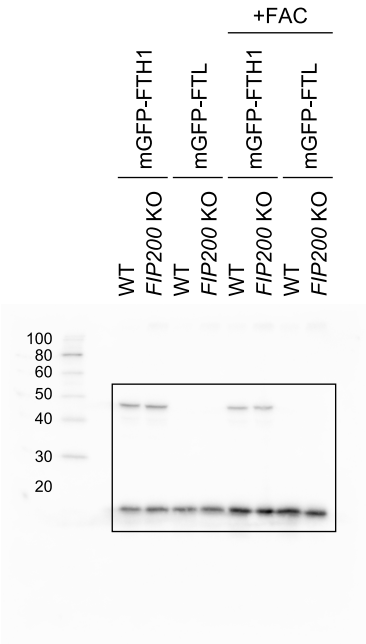

anti-NCOA4 (Figure 1C)

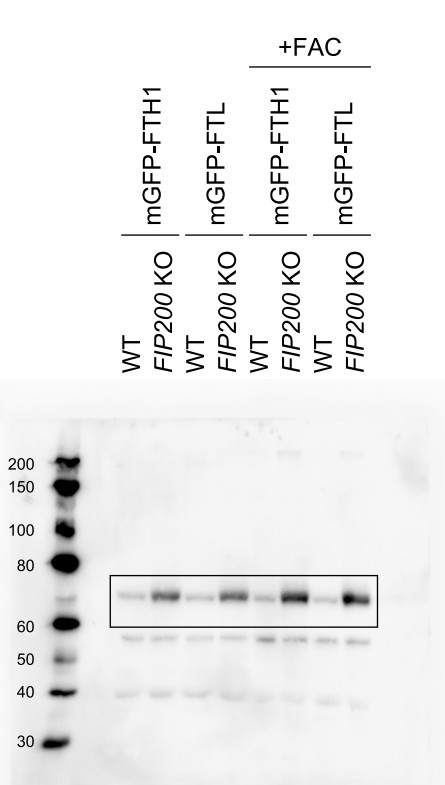

anti-FTL (Figure 1C)

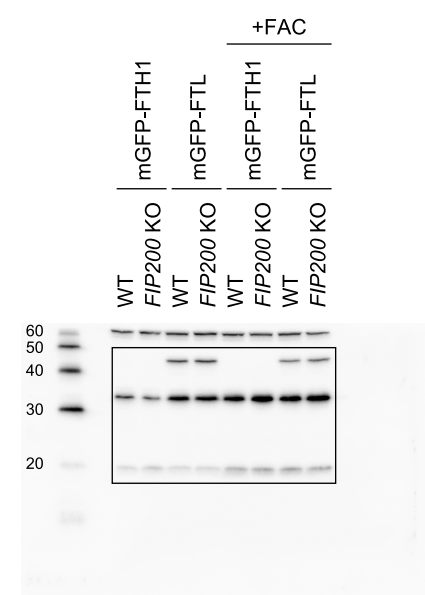

anti-TAX1BP1 (Figure 1C)

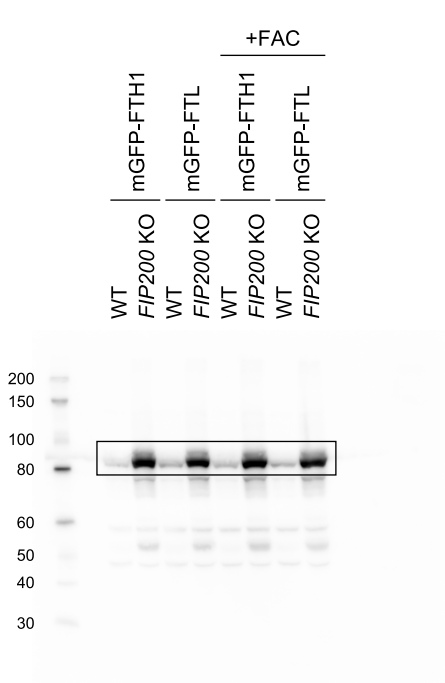

anti-HSP90 (Figure 1C)

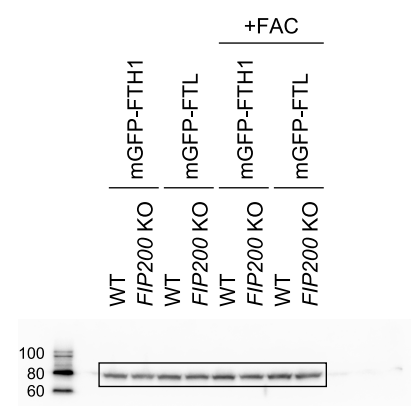

Supplement: SourceData F1 — is the source file for Fig. 1. [file JCB_202203102_SourceDataF1.pdf]

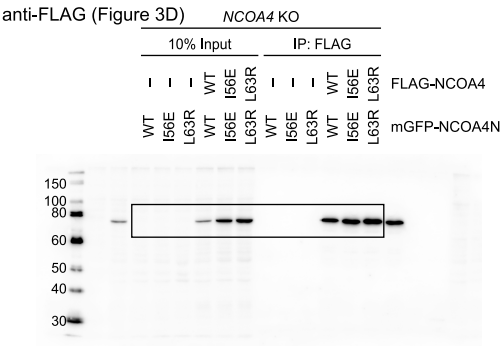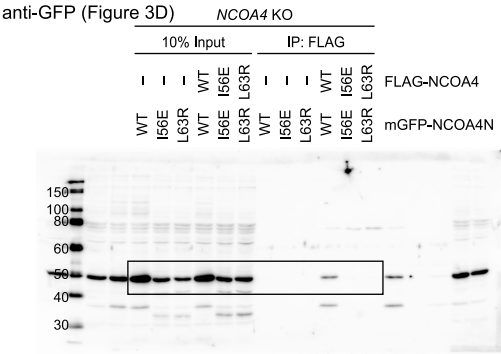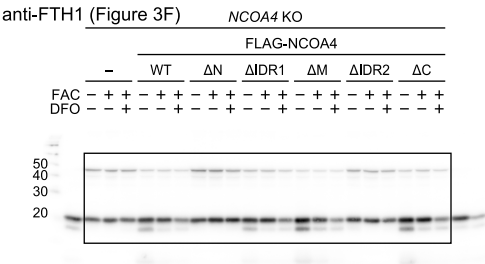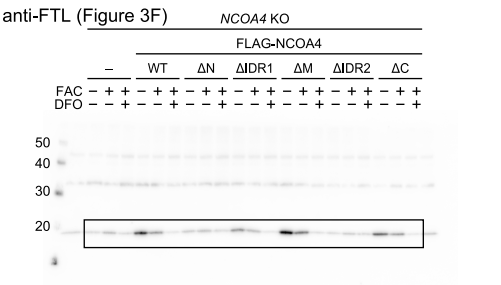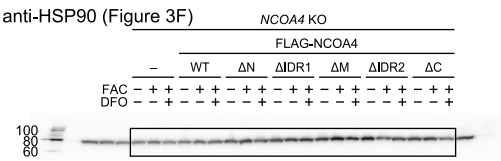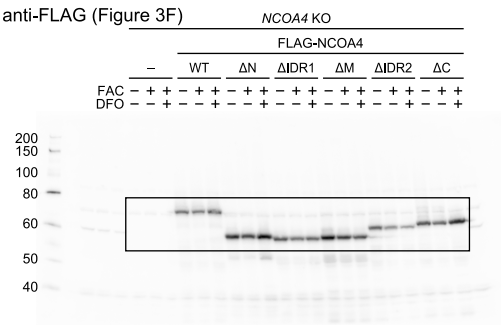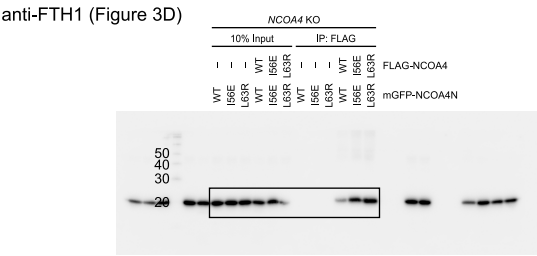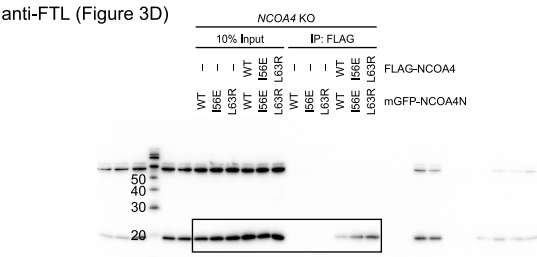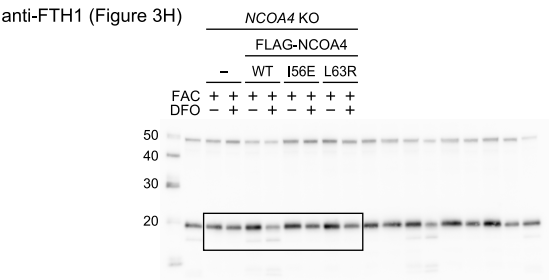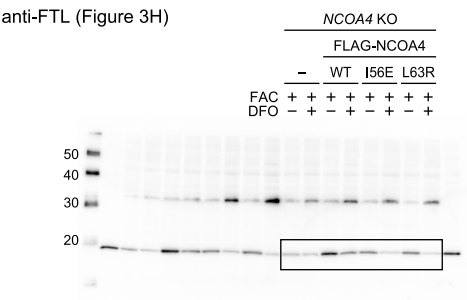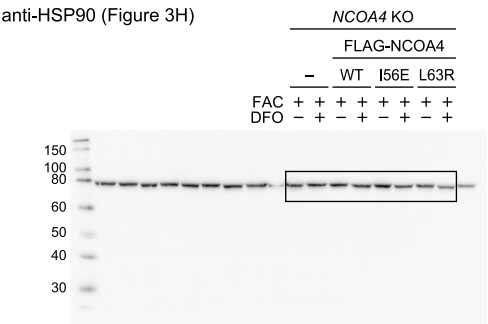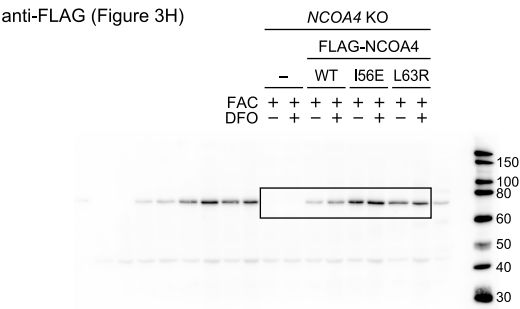

SourceDataF3. Uncropped images used for Figures 3D, 3F, 3H

Supplement: SourceData F3 — is the source file for Fig. 3. [file JCB_202203102_SourceDataF3.pdf]
