# Supplementary material for: NCOA4 drives ferritin phase separation to facilitate macroferritinophagy and microferritinophagy
Source: J Cell Biol. 2022 Sep 6;221(10):e202203102. doi: 10.1083/jcb.202203102 (PMC9452830; doi:10.1083/jcb.202203102)
Supplement: SourceData F2 — is the source file for Fig. 2. [file JCB_202203102_SourceDataF2.pdf]

anti-FTH1 (Figure 2E)

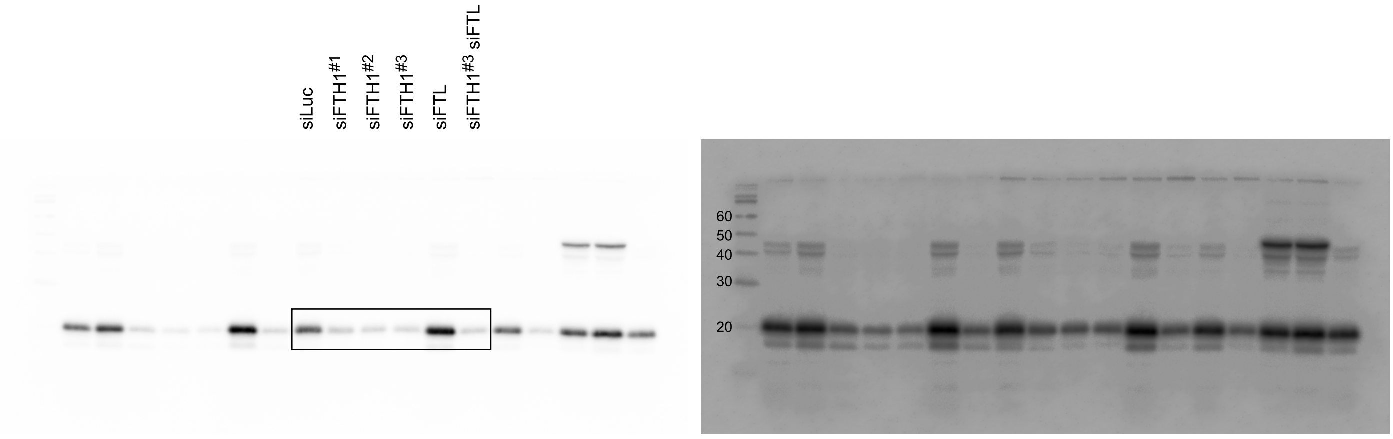

anti-FTL (Figure 2E)

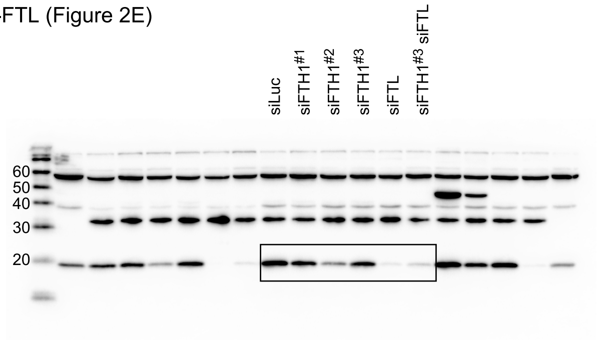

anti-NCOA4 (Figure 2E)

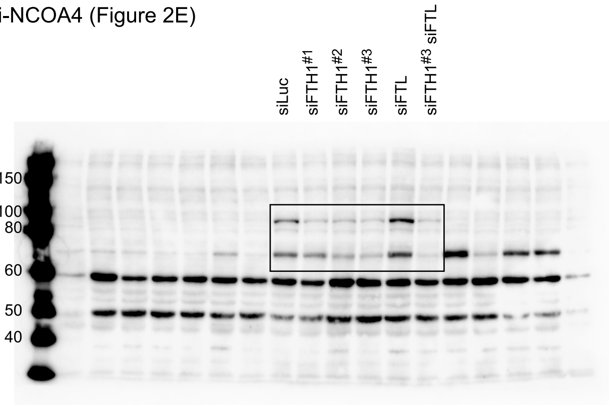

anti-HSP90 (Figure 2E)

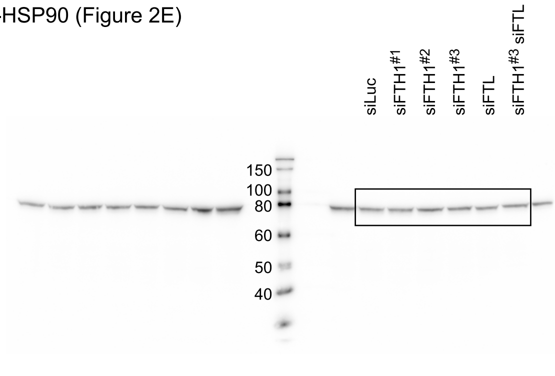

SourceDataF2. Uncropped images used for Figure 2E
