# Supplementary material for: NCOA4 drives ferritin phase separation to facilitate macroferritinophagy and microferritinophagy
Source: J Cell Biol. 2022 Sep 6;221(10):e202203102. doi: 10.1083/jcb.202203102 (PMC9452830; doi:10.1083/jcb.202203102)
Supplement: SourceData FS1 — is the source file for Fig. S1. [file JCB_202203102_SourceDataFS1.pdf]

anti-NCOA4 (Figure S1E)

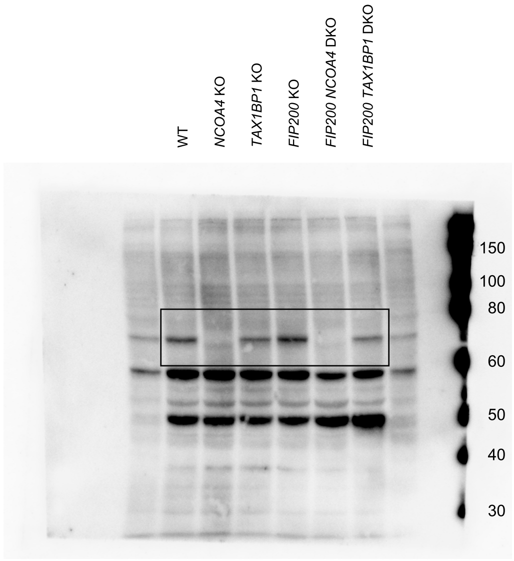

anti-FTH1 (Figure S1F)

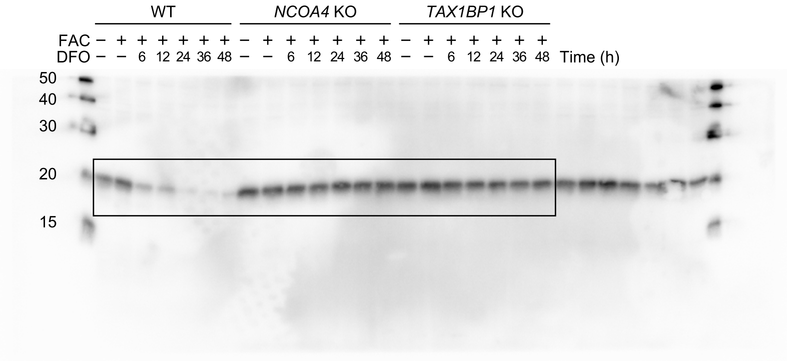

anti-HSP90 (Figure S1F)

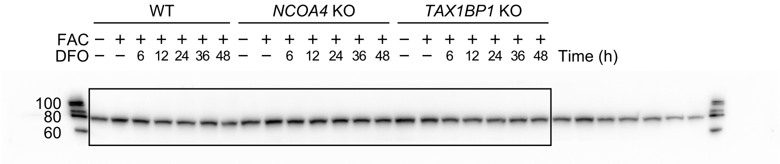

anti-TAX1BP1 (Figure S1E)

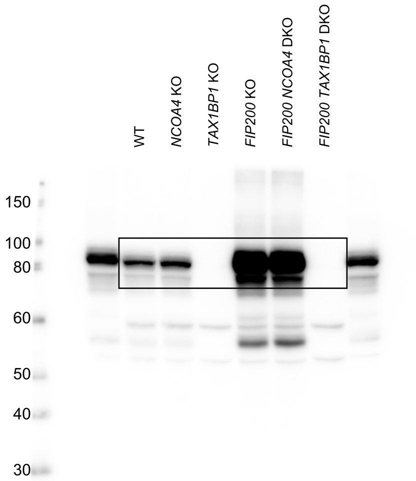

anti-HSP90 (Figure S1E)

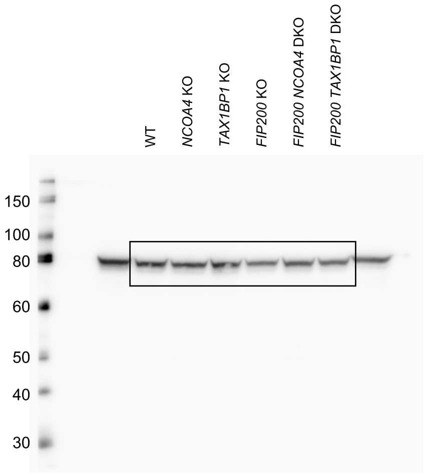

anti-FIP200 (Figure S1E)

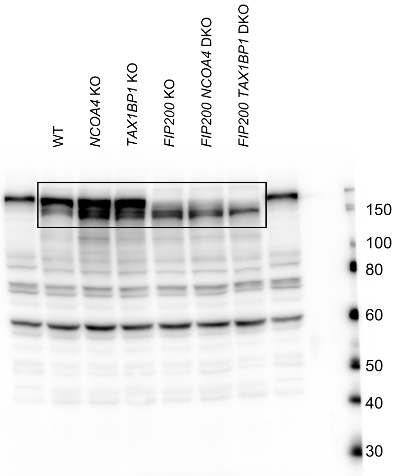

SourceDataFS1. Uncropped images used for Figures S1E, S1F
